# Quantitative assessment of systolic left ventricular function with speckle-tracking echocardiography in adult patients with repaired aortic coarctation

**DOI:** 10.1007/s10554-016-0838-8

**Published:** 2016-01-16

**Authors:** Myrthe E. Menting, Roderick W. J. van Grootel, Annemien E. van den Bosch, Jannet A. Eindhoven, Jackie S. McGhie, Judith A. A. E. Cuypers, Maarten Witsenburg, Willem A. Helbing, Jolien W. Roos-Hesselink

**Affiliations:** Department of Cardiology, Erasmus MC, P.O. Box 2040, 3000 CA Rotterdam, The Netherlands; Division of Pediatric Cardiology, Department of Pediatrics, Erasmus MC - Sophia Children’s Hospital, P.O. Box 2060, 3000 CB Rotterdam, The Netherlands; Department of Cardiology, Erasmus MC, Room Ba-583a, ‘s-Gravendijkwal 230, 3015 CE Rotterdam, The Netherlands

**Keywords:** Aortic coarctation, Left ventricular function, Myocardial deformation, Speckle-tracking echocardiography, Strain

## Abstract

Despite successful aortic coarctation (CoA) repair, systemic hypertension often recurs which may influence left ventricular (LV) function. We aimed to detect early LV dysfunction using LV global longitudinal strain (GLS) in adults with repaired CoA, and to identify associations with patient and echocardiographic characteristics. In this cross-sectional study, patients with repaired CoA and healthy controls were recruited prospectively. All subjects underwent echocardiography, ECG and blood sampling within 1 day. With speckle-tracking echocardiography, we assessed LV GLS on the apical four-, three- and two-chamber views. We included 150 subjects: 75 patients (57 % male, age 33.4 ± 12.8 years, age at repair 2.5 [IQR: 0.1–11.1] years) and 75 healthy controls of similar sex and age. LV GLS was lower in patients than in controls (−17.1 ± 2.3 vs. −20.2 ± 1.6 %, *P* < 0.001). Eighty percent of the patients had a normal LV ejection fraction, but GLS was still lower than in controls (*P* < 0.001). In patients, GLS correlated with systolic and diastolic blood pressure (r = 0.32, *P* = 0.009; r = 0.31, *P* = 0.009), QRS duration (r = 0.34, *P* = 0.005), left atrial dimension (r = 0.27, *P* = 0.029), LV mass (r = 0.30, *P* = 0.014) and LV ejection fraction (r = −0.48, *P* < 0.001). Patients with either associated cardiac lesions, multiple cardiac interventions or aortic valve replacement had lower GLS than patients without. Although the majority of adults with repaired CoA seem to have a normal systolic LV function, LV GLS was decreased. Higher blood pressure, associated cardiac lesions, and larger left atrial dimension are related with lower GLS. Therefore, LV GLS may be used as objective criterion for early detection of ventricular dysfunction.

**Electronic supplementary material:**

The online version of this article (doi:10.1007/s10554-016-0838-8) contains supplementary material, which is available to authorized users.

## Introduction

Coarctation of the aorta (CoA) is common (5–8 % of all congenital heart defects) and is considered to be part of a generalized arteriopathy with a reduced compliance of arterial vascular walls, instead of only a circumscript narrowing of the aorta [1–3]. Patients can have associated lesions, such as a bicuspid aortic valve, subvalvular, valvular, or supravalvular aortic stenosis, and mitral valve stenosis [3]. CoA causes left ventricular (LV) pressure overload, which can lead to increased myocardial wall stress, LV systolic and diastolic dysfunction, and the development of arterial collaterals [3]. In order to relieve the obstruction, a surgical or transcatheter intervention is needed. Despite successful repair, late cardiovascular problems occur including systemic hypertension in 30–75 % of the cases [4–6], compensatory LV hypertrophy, heart failure, coronary heart disease, stroke and sudden cardiac death [6, 7]. Early detection of LV dysfunction could be important for risk stratification or early initiation of treatment. Speckle-tracking echocardiography (STE) is a sophisticated technique that provides a quantitative assessment of the motion of myocardial tissue, independently of angle and ventricular geometry, which could detect subclinical myocardial dysfunction [8, 9]. One of the measurements is strain imaging which is defined as deformation of the myocardial wall normalized to its original size. Because strain in longitudinal direction is the most widely used type of strain and is a robust index for clinical studies [10], we have chosen to focus on longitudinal strain. One study recently described a significant lower LV global longitudinal strain (GLS) after CoA repair in a small cohort of heterogeneous patients including children and adults [11]. Our group of CoA subjects, age- and sex-matched, is the largest and oldest in which LV GLS has been studied to date.

The aim was to evaluate LV GLS in adults after CoA repair and healthy controls of similar age and sex, and to study relationships between GLS and patient characteristics such as smoking, interventions, hypertension, aortic valve morphology, other associated congenital cardiac lesions, and prior cardiovascular events, but also with conventional echocardiographic measurements and cholesterol and N-terminal pro-Brain Natriuretic Peptide (NT-proBNP) levels.

## Methods

### Study population

Consecutive patients who were ≥18 years of age and had undergone CoA repair were recruited at the adult outpatient cardiology clinic at Erasmus MC between September 2011 and June 2014. Exclusion criteria were a pacemaker, irregular heart rhythm, and poor quality of the echocardiographic images at all apical views for adequate STE. This prospective study’s protocol included medical history, physical examination, echocardiography, 12-lead electrocardiography, and cholesterol and NT-proBNP measurements all on the same day. Patient characteristics included age, sex, medication, clinical parameters, type of initial repair, number of interventions (surgical or transcatheter), aortic valve replacement, aortic valve morphology, other associated congenital cardiac lesions and prior cardiovascular events (coronary artery disease, heart failure, stroke). Hypertension was defined as the requirement of antihypertensive drugs or when ≥3 times an elevated blood pressure was measured (systolic >140 mmHg or diastolic >90 mmHg). Healthy controls of similar age and sex were voluntarily recruited via an advertisement. They had no medical history or current symptoms suggesting cardiovascular disease and did not take any chronic medication.

### Echocardiographic image acquisition

Two-dimensional greyscale harmonic images were obtained in the left lateral decubitus position using an iE33 ultrasound system (Philips Medical Systems, Best, The Netherlands) equipped with a transthoracic X5-1 matrix transducer (composed of 3040 elements, with 1–5 MHz extended operating frequency range). Images were acquired at frame rates of >55 frames/s. The echocardiographic studies were stored in digital imaging and communications in medicine (DICOM) format.

### Conventional echocardiographic measurements

For chamber measurements and LV mass calculation, we used the current recommendations for cardiac chamber quantification [12]. For normal systolic LV ejection fraction (EF) assessed with the Simpson’s method, we used the reference values of ≥52 % for males and ≥54 % for females [12]. In addition, LV systolic function was visually graded as normal or mildly, moderately or severely impaired. From the apical four-chamber view (A4C), pulsed-wave Doppler examination was performed to obtain peak mitral inflow velocities at early (E) and late (A) diastole and E deceleration time. Tissue Doppler imaging was performed to obtain myocardial tissue velocity at the mitral annulus at early diastole (E′). For left atrial (LA) size, we measured the anteroposterior diameter in the parasternal long-axis view and the LA area in the A4C at end-systole [12]. For the assessment and grading of valvular stenosis and regurgitation, we used the recommendations of the European Association of Echocardiography [13–15].

### Speckle-tracking analysis

Offline analysis of the data sets was performed using STE by QLAB version 9.0 (Philips Medical Systems, Best, The Netherlands). Analysis was performed according to the vendor’s instructions. Cardiac cycles were defined by the positioning of R-waves.

**Fig. 1:**
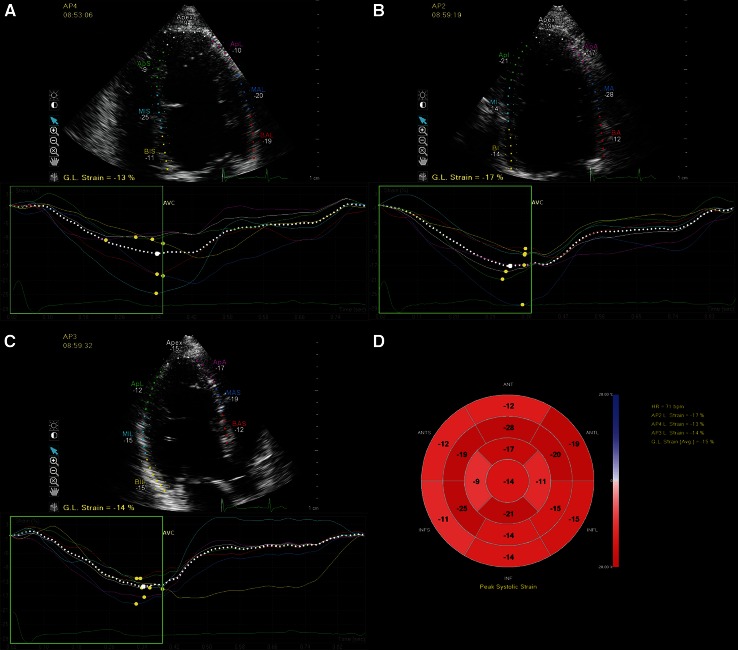
Example of left ventricular longitudinal strain measurements in a patient with repaired aortic coarctation. The LV was traced in the apical four-, two-, and three-chamber views at end-diastole. The walls were automatically divided into seven segments at each view and the global longitudinal strain at each view was calculated (**a**–**c**). The segmental strain measurements were plotted in a bull’s eye and the left ventricular global longitudinal strain based on all three apical views was calculated (**d**). *AP2* apical two-chamber view, *AP3* apical three-chamber view, *AP4* apical four-chamber view, *GL* global longitudinal

To assess peak systolic LV GLS, the endocardial and epicardial borders were traced in the apical four-, three- and two-chamber views (A3C, A2C) on an end-diastolic frame (Fig. 1a–c). The software automatically divided the walls in several segments (LV algorithm based on 17-segment model) and tracked these points on a frame-by-frame basis. When tracking was suboptimal, we readjusted the borders. Segments with persistently inadequate tracking were excluded from further analysis. Peak systolic strain values were defined as the peak values on the curves during the ejection phase. We reported the peak systolic LV GLS based on measurements of all three apical views (Fig. 1d). Data were exported to a spreadsheet program (Excel; Microsoft Corporation, Redmond, WA, USA). All references to strain changes consider the absolute value of the number, so that higher or increase in longitudinal strain means a more negative number and lower or decrease means a less negative number [8].

### NT-proBNP measurement

Peripheral venous blood samples were collected and plasma NT-proBNP levels were determined with use of the commercially available electrochemiluminescence immunoassay Elecsys (Roche Diagnostics, Basel, Switzerland). The normal value in our hospital is <14 pmol/L.

### Statistical analysis

Continuous variables are presented as mean ± standard deviation (SD) or as median with interquartile range [Q1–Q3]. Categorical variables are presented as frequencies and percentages. For comparison of normally distributed continuous variables between two groups the Student’s *t* test was used and in case of skewed distribution, the Mann–Whitney *U* test. For comparison between three groups the Kruskal–Wallis sum test was applied. For comparison of frequencies, the χ^2^-test or Fisher’s exact test was used. For quantifying correlations between two variables, the Pearson or Spearman correlation test was applied. Multivariable regression analysis was performed for patient characteristics which were significant associated with LV GLS. In case of collinearity of these variables, we implemented the variable with the strongest correlation into multivariable analysis. Because NT-proBNP values were not normally distributed, the values were log transformed for further statistical analyses.

Intra-observer and inter-observer agreement between two investigators (MM, RvG) were assessed by repeated analysis in two third of the data sets at least 2 months after the initial analysis on the second cardiac cycle at the same images and blinded to the initial results. The limits of agreement between two measurements were determined as the mean of the differences ± 1.96 SD and presented in a Bland–Altman plot [16]. Additionally, the coefficient of variation (COV; SD of the differences of two measurements divided by their mean) was provided.

All statistical analyses were performed using SPSS statistics version 21 (IBM Corp., Armonk, NY, USA). The statistical tests were two-sided and a *P* < 0.05 was considered statistically significant.

## Results

### Study population

**Fig. 2:**
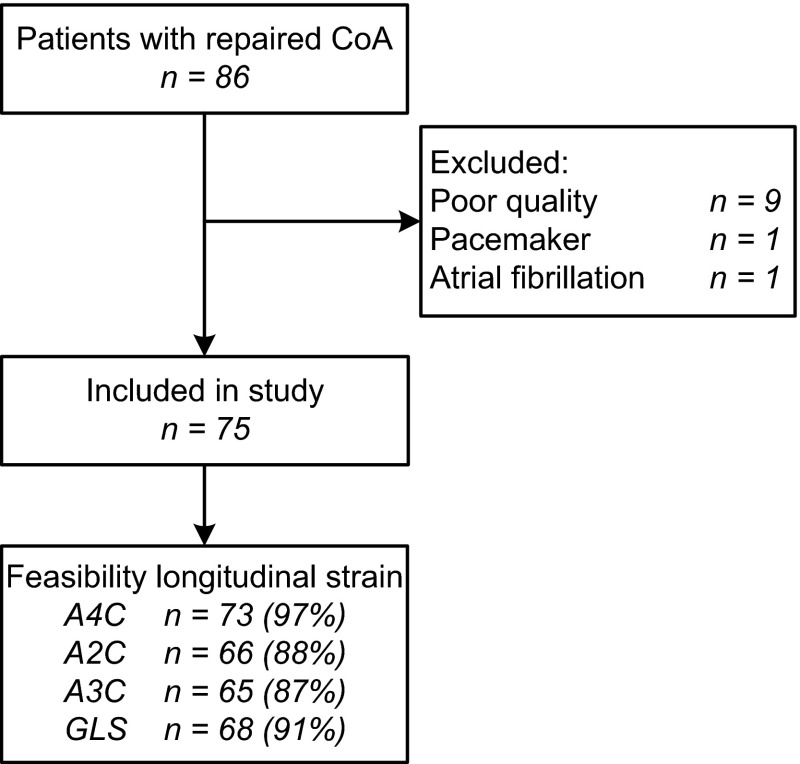
Flow chart of the study patients. An overview of the patient inclusion and feasibility of the left ventricular longitudinal strain measurements at the different apical views. *A2C* apical two-chamber view, *A3C* apical three-chamber view, *A4C* apical four-chamber view, *GLS* global longitudinal strain

We included 75 adult patients (57 % male, age 33.4 ± 12.8 years) and 75 healthy controls of similar sex and age. Figure 2 presents an overview of the patient participation and feasibility of the measurements. Table 1 shows the characteristics of the study population. The median age at the initial CoA repair, surgical or transcatheter, was 2.5 years [0.1–11.0; range: 0–51] and the median follow-up after repair was 24.7 years [20.4–31.7]. Sixteen patients underwent aortic valve replacement 11.6 years [5.4–18.0] prior to this study. Six patients (8 %) had a history of at least one cardiovascular event: transient ischemic attack (n = 3), postoperative cerebrovascular accident after aortic valve replacement (n = 1), subarachnoidal bleeding (n = 1), and percutaneous coronary intervention because of progressive angina (n = 1). Of the 20 patients with QRS duration >120 ms, 8 had a right bundle branch block, 6 a left, and 6 unspecified. Only one patient (1 %) had an elevated cholesterol level of 7.7 mmol/L.

**Table 1:** Characteristics of the study population

Clinical characteristics	CoA patients (n = 75)	Healthy controls (n = 75)	*P* value
Age at time of study (years)	33.4 ± 12.8	33.9 ± 10.6	0.793
Female	32 (43 %)	32 (43 %)	1.000
BMI (kg/m^2^)	25.0 ± 4.1	23.4 ± 3.2	0.015
BSA (m^2^)	1.90 ± 0.22	1.88 ± 0.16	0.675
Systolic blood pressure (mmHg)	132 ± 17	125 ± 13	0.007
Diastolic blood pressure (mmHg)	79 ± 12	76 ± 9	0.194
Smoker (current or former)	6 (8 %)	2 (3 %)	1.0
Hypertension^a^	38 (51 %)	0 (0 %)	<0.001
Any antihypertensive drugs	33 (44 %)	0 (0 %)	<0.001
Betablocker	15 (20 %)	–	–
ACE inhibitor	13 (17 %)	–	–
Angiotensin II antagonist	10 (13 %)	–	–
Diuretics	7 (9 %)	–	–
Aldosteron antagonist	2 (3 %)	–	–
NYHA functional Class I/Class II	74 (99 %)/1 (1 %)	75 (100 %)/0	–
Heart rate (bpm)	63 ± 11	64 ± 11	0.701
QRS duration (ms)	113 ± 19	97 ± 10	<0.001
QRS duration >120 ms	20 (27 %)	0	–
Age at initial repair (years)	2.5 [0.1–11.0]	–	–
Type of initial repair			
End-to-end anastomosis	53 (70 %)	–	–
Teflon patch aortoplasty	9 (12 %)	–	–
Subclavian flap aortoplasty	8 (11 %)	–	–
Bypass	3 (4 %)	–	–
Stent	2 (3 %)	–	–
Total number of cardiac interventions			
One	35 (47 %)	–	–
Two	21 (28 %)	–	–
Three or more	19 (25 %)	–	–
Repeated coarctation repair	25 (33 %)	–	–
Aortic valve replacement	16 (21 %)	–	–
Native aortic valve morphology			
Bicuspid	52 (70 %)	–	–
Tricuspid	21 (28 %)	–	–
Unknown	2 (2 %)	–	–
Other congenital cardiac lesions^b^	33 (44 %)	–	–
Ventricular septal defect	20 (27 %)	–	–
Patent ductus arteriosus	11 (15 %)	–	–
PAPVR	3 (4 %)	–	–
Other	9 (12 %)	–	–
Cholesterol level (mmol/L)	4.9 [4.0–5.5]	–	–
NT-proBNP (mmol/L)	7.2 [3.6–16.7]		

Categorical data are presented as n (%) and continuous data as mean ± SD or median [interquartile range]

*ACE* angiotensin converting enzyme, *BMI* body mass index, *BSA* body surface area, *NYHA* New York Heart Association, *PAPVR* partial anomalous pulmonary venous return

^a^Requiring antihypertensive drugs or ≥3 times measured elevated blood pressure (systolic >140 mmHg or diastolic >90 mmHg)

^b^Apart from bicuspid aortic valve

Conventional echocardiographic measurements of all subjects are presented in Table 2. All diastolic measurements in the CoA patients, except for deceleration time, were significantly different from normal controls. Visually assessed systolic LV function was graded normal in 63 (84 %) patients, mildly impaired in 11 (15 %), and severely impaired in 1 (1 %). The diastolic function was graded as a normal pattern in 54 (72 %) patients, abnormal relaxation pattern in 3 (4 %), pseudonormal filling pattern in 9 (12 %), restrictive pattern in 5 (7 %), and in 4 (5 %) patients the diastolic function was not analyzable. Aortic stenosis (>2.5 m/s) was observed in 13 (17 %) patients. Aortic regurgitation was graded mild in 32 (43 %), moderate in 3 (4 %) and severe in none of the patients. Mitral regurgitation was graded mild in 20 (27 %), moderate in 3 (4 %) and severe in none. Tricuspid regurgitation could be measured in 51 patients, of whom 3 (6 %) had a peak velocity of >2.8 m/s.

**Table 2:** Conventional echocardiographic measurements of the study patients

Echocardiographic measurements	Patients (n = 75)	Controls (n = 75)	*P* value
LA dimension (mm)	35 ± 6	33 ± 4	0.124
LV end-diastolic dimension (mm)	50 ± 5	48 ± 4	0.034
LV end-systolic dimension (mm)	31 ± 5	29 ± 3	0.010
Interventricular septum (mm)	9.7 ± 2.7	8.3 ± 1.5	<0.001
LV posterior wall (mm)	9.0 ± 1.5	8.6 ± 1.2	0.052
LV mass (g)	169 ± 56	136 ± 30	<0.001
LV EF Simpson’s (%)	57 ± 7	64 ± 4	<0.001
LV E wave (m/s)	1.02 ± 0.25	0.76 ± 0.14	<0.001
LV A wave (m/s)	0.69 ± 0.23	0.43 ± 0.11	<0.001
LV E/A ratio	1.58 ± 0.54	1.90 ± 0.57	0.001
LV deceleration time (ms)	207 ± 62	193 ± 32	0.098
LV E′ (cm/s)	9.0 ± 2.5	11.2 ± 2.8	<0.001
LV E/E′ ratio	12.4 ± 5.9	7.1 ± 1.8	<0.001

Data are presented as mean ± SD

*A* peak mitral inflow velocity at late diastole, *E* peak mitral inflow velocity at early diastole, *E*′ early diastolic annular myocardial velocity, *EF* ejection fraction, *LA* left atrium, *LV* left ventricle

### Left ventricular global longitudinal strain

**Fig. 3:**
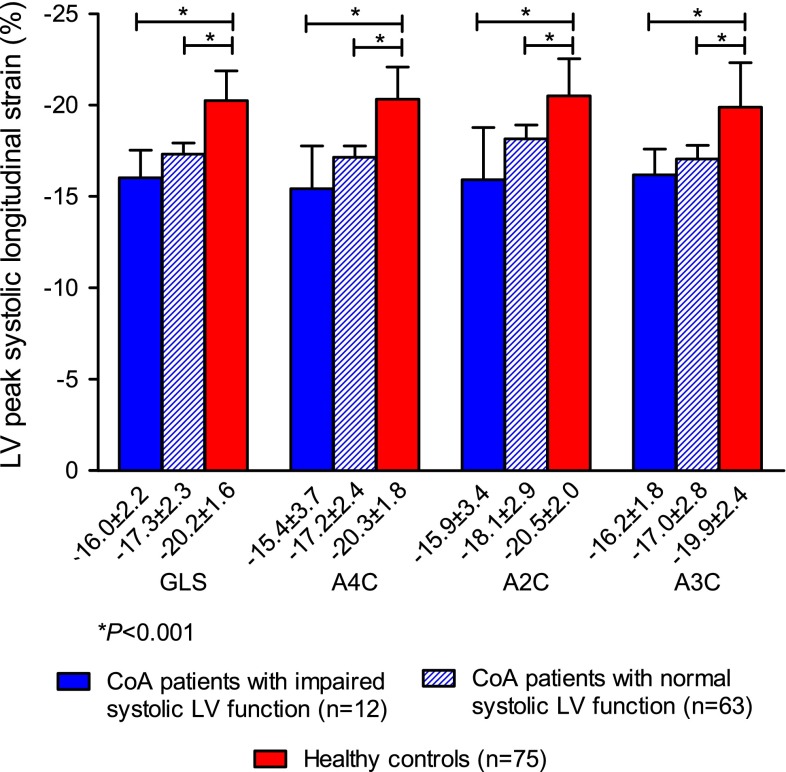
Left ventricular global longitudinal strain in CoA patients with visually graded impaired and normal left ventricular systolic function and in healthy controls. This figure presents the mean peak systolic LV GLS based on the measurements in the apical four-, two- and three-chamber view, and the mean peak systolic longitudinal strain at these three views separately. Strain values of the patients with visually graded normal left ventricular systolic function and of patients with impaired left ventricular systolic function are both compared with those of healthy controls. *A2C* apical two-chamber view, *A3C* apical three-chamber view, *A4C* apical four-chamber view, *GLS* global longitudinal strain, *LV* left ventricular

Patients had a significantly lower mean GLS based on all three apical views (−17.1 ± 2.3 %), GLS on A4C (−16.9 ± 2.7 %), GLS on A2C (−17.9 ± 3.0 %), and GLS on A3C (−16.9 ± 2.4 %) than the healthy controls (all *P* < 0.001). A sub analysis in the 63 patients (84 %) with a visually graded normal systolic LV function revealed that GLS was still significantly lower than in controls (Fig. 3). LV EF was measurable in 49 patients of whom 39 (80 %) had a normal EF. A sub analysis in these 39 patients, also showed that GLS in all three apical views separately was significantly lower than in controls (all *P* < 0.001).

### Relationships with patient characteristics

**Fig. 4:**
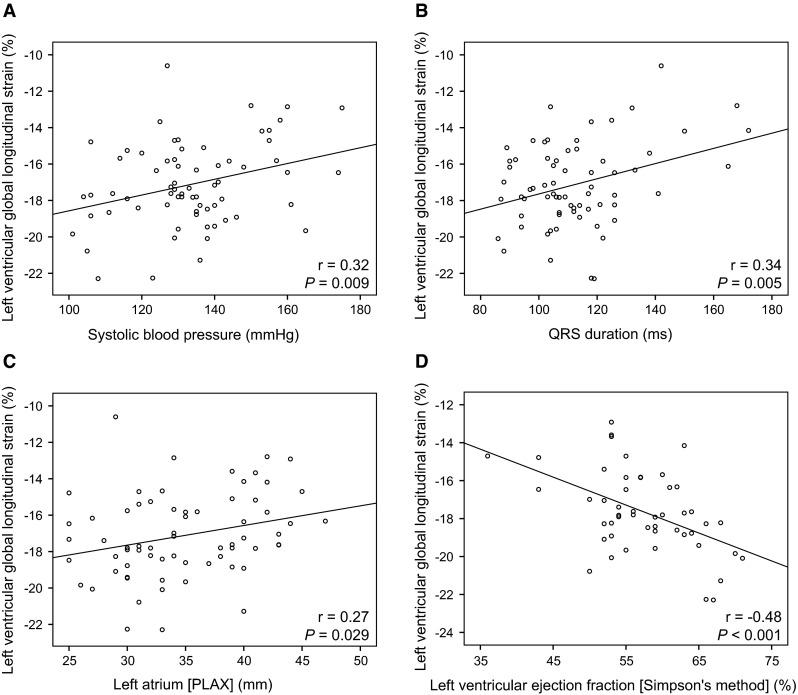
Scatter plots showing correlations with left ventricular global longitudinal strain. Significant correlations were observed between LV GLS and systolic blood pressure (**a**), QRS-duration (**b**), left atrial dimension at parasternal long axis view (**c**), and left ventricular ejection fraction measured with Simpson’s biplane method (**d**)

**Fig. 5:**
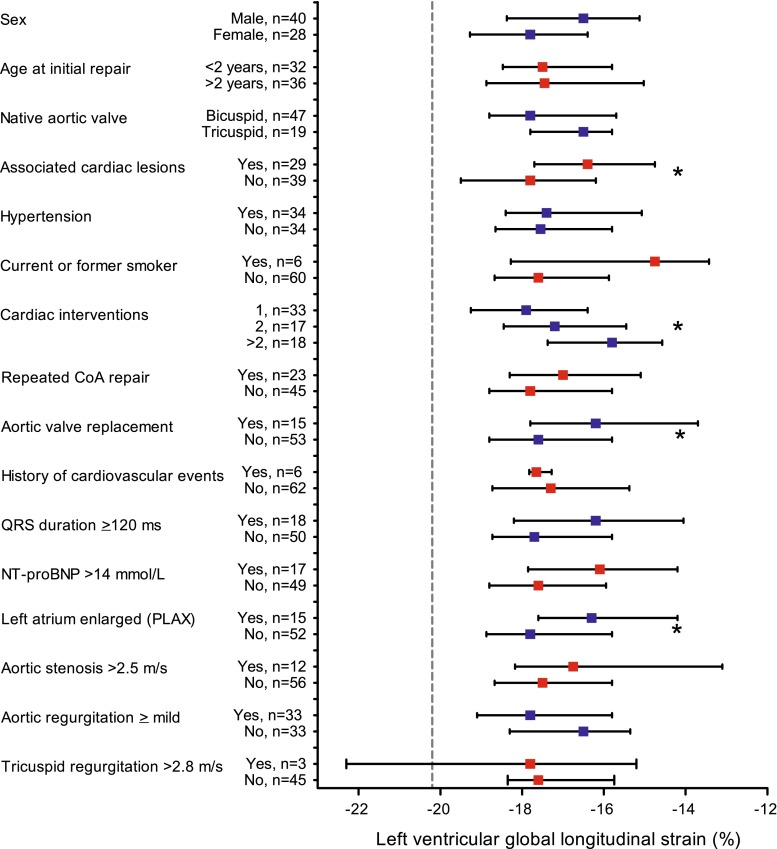
Comparisons of left ventricular global longitudinal strain in CoA patients sorted in various groups. Symbols present median and whiskers present interquartile range. The *dashed grey line* depicts the mean LV global longitudinal strain of the healthy controls. *P < 0.05. *PLAX* parasternal long axis view

Table 3 present the relationships between LV GLS and patient characteristics. Patients with higher BMI, higher blood pressure or longer QRS duration had a lower GLS in all three apical views (Fig. 4a, b). Patients who had one or more associated congenital cardiac lesions, who had undergone multiple cardiac interventions or who had undergone aortic valve replacement had a significantly lower GLS than patients without these interventions (Fig. 5). No significant associations were found between GLS and current age, age at repair or repeated CoA repair.

**Table 3:** Correlations with left ventricular global longitudinal strain

	Correlation coefficient	*P* value
*Patient characteristics*		
Age^a^	0.18	0.132
Age at repair^a^	0.04	0.759
BMI^a^	**0.29**	**0.018**
Systolic blood pressure	**0.32**	**0.009**
Diastolic blood pressure	**0.31**	**0.009**
QRS duration	**0.34**	**0.005**
Cholesterol level	0.11	0.396
Ln NT-proBNP	0.11	0.393
*Echocardiographic measurements*		
LA dimension	**0.27**	**0.029**
LV end-diastolic dimension	0.08	0.509
LV end-systolic dimension	−0.02	0.889
Interventricular septum^a^	**0.33**	**0.008**
LV posterior wall^a^	**0.38**	**0.002**
LV mass^a^	**0.30**	**0.014**
LV EF Simpson’s	−**0.48**	**<0.001**
LV E wave	−0.23	0.058
LV A wave^a^	−0.03	0.815
LV E/A ratio^a^	−0.20	0.117
LV deceleration time^a^	−0.05	0.673
LV E′	−0.19	0.149
LV E/E′ ratio^a^	−0.13	0.341

Bold represents statistically significant differences

*A* peak mitral inflow velocity at late diastole, *BMI* body mass index, *E* peak mitral inflow velocity at early diastole, *E*′ early diastolic annular myocardial velocity, *EF* ejection fraction, *LA* left atrium, *Ln* = natural logarithm, *LV* left ventricle, *NT-proBNP* N-terminal pro-brain natriuretic peptide

^a^Spearman’s correlation coefficient

Multivariable regression analysis with the variables ‘systolic blood pressure’, ‘associated cardiac lesions’ and ‘aortic valve replacement’ revealed that the systolic blood pressure and the presence of associated cardiac lesions were independently associated with LV GLS (β = 0.290, *P* = 0.009; β = 0.353, *P* = 0.002, respectively), and that a trend was seen regarding an aortic valve replacement (β = 0.188, *P* = 0.087). Other significant variables were not implemented in the multivariable analysis because of collinearity between the variables. Patients without comorbidity such as associated cardiac lesions, aortic valve replacement, cardiac reinterventions or hypertension (n = 13) still had a lower GLS than the healthy controls (*P* = 0.001).

In patients with elevated NT-proBNP, GLS on A4C was significantly lower (*P* = 0.010) and GLS based on all three apical views tended to be significantly lower (*P* = 0.057).

### Relationships with conventional echocardiographic measurements

Table 3 summarises the relationships between LV GLS and echocardiographic measurements. Patients with a higher LV mass, lower LV EF or larger LA dimension had a lower GLS based on all three apical views (Fig. 4c, d). LA area did not correlate with GLS. A higher E wave was associated with a higher GLS on A4C (r = −0.25, *P* = 0.035) and a trend was found towards higher GLS based on all three apical views. Online Resource 1 presents the clinical and echocardiographic characteristics stratified by tertiles of GLS values.

### Intra-observer and inter-observer agreement

**Fig. 6:**
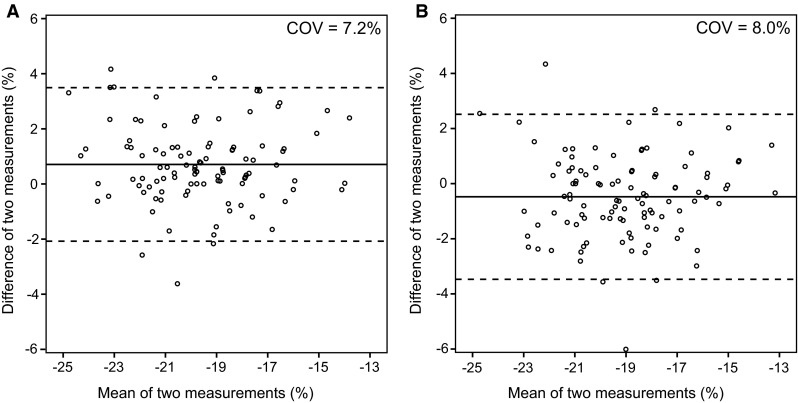
Bland–Altman plots demonstrating intra-observer and inter-observer agreement. Left ventricular global longitudinal strain measurements on apical four-chamber view for intra-observer (**a**) and inter-observer agreement (**b**). The *solid lines* depict the mean difference of two measurements, and the *dashed lines* depict the limits of agreement. *COV* coefficient of variation

The intra-observer agreement for the GLS on A4C was 0.71 ± 1.42 % with a COV of 7.2 %. The inter-observer agreement was −0.48 ± 1.53 % with a COV of 8.0 %. Figure 6 depicts the Bland–Altman plots.

## Discussion

This prospective study shows that LV GLS is reduced in patients late after CoA repair, providing evidence of subclinical LV dysfunction, which is not detectable with conventional 2D-echocardiography. Although survival of CoA patients have been improved since the introduction of cardiac surgery and percutaneous interventions, morbidity after CoA repair is still substantial. More than one third of these patients encounter late cardiovascular complications after repair. However, these results are from the early years of cardiac surgery [7]. In order to reduce morbidity, detection of early ventricular dysfunction may identify patients at risk of developing ventricular failure or adverse cardiac events. LV GLS could probably be a useful prognostic follow-up tool in these patients.

### Decreased left ventricular global longitudinal strain

The feasibility of GLS was higher than the feasibility of EF by Simpson’s method. This could be explained by the fact that with STE speckles are followed instead of tracing the endocardial border by Simpson’s. The last method requires higher quality images. Another important finding is that adult patients after CoA repair have decreased LV GLS while their EF is measured as normal. This was also observed in a recent study with CMR feature tracking [17]. Older age at repair has been shown as a predictor for LV long-axis dysfunction [18], however, even in children with CoA repair, a decreased GLS was already observed whereas the EF was normal [19]. In our cohort, patients more often had impaired LV diastolic function than controls, which is in line with the observed abnormal LV diastolic function in children with CoA [20]. Lombardi et al. found a strong correlation between proximal ascending aortic elasticity and diastolic function in all children and suggested that, although CoA repair was successfully performed, persistently elevated aortic stiffness may lead to diastolic impairment. Arterial stiffness is a risk factor for cardiovascular events and mortality, and is associated with aging, hypertension and systemic disorders [21]. The relationship between arterial stiffness and intima media thickness with LV systolic and diastolic deformation has also been described in adults after CoA repair [11] and corresponds with our finding that blood pressure correlates with LV deformation. A meta-analysis of possible demographic and hemodynamic variables that contribute to LV GLS in healthy subjects showed that only blood pressure was independently associated with strain values [22]. However, even in the patient group without hypertension, we still observed a decreased GLS compared with controls suggesting that other factors apart from blood pressure have impact on LV GLS. Besides the arterial stiffness, GLS could also be affected by myocardial fibrosis or by inflammation [23, 24]. The observed chronic inflammatory and possibly apoptotic reaction in adults with repaired CoA reflects a functional problem in all vessels, regardless of the initial lesion. This could be implied by the increased levels of circulated cytokines specifically related to vascular endothelial dysfunction [24]. All these factors may explain why CoA patients have an increased risk of developing late cardiovascular complications despite early repair and improved surgical procedures. To state whether decreased strain really enables the detection of preclinical LV dysfunction, follow-up studies are warranted in both children and adults. In addition, studies are needed to determine whether it is possible with the use of strain to distinguish between patients who will and who will not benefit from early treatment to reduce morbidity.

### Relationships with patient characteristics

Univariable regression analysis showed that patients who had associated congenital cardiac lesions, who underwent multiple cardiac interventions or who underwent aortic valve replacement had a more decreased GLS. This adverse interaction could partly be explained by the interventions themselves and probably by the long-time exposure to volume or pressure overload. In contrast, we did not find a significant difference in strain between patients with one CoA repair versus repeated CoA repair. Higher current blood pressure is related to lower GLS in our patient group. However, there is no significant difference in GLS between patients who met the criteria for hypertension and those who did not. This could possibly be explained by the antihypertensive drugs used by these patients resulting in a normal blood pressure nowadays. These findings stress the importance of tight blood pressure regulation in this patient population even after successful CoA repair. The effect of tight blood-pressure control on GLS in these patients may be of interest and deserves further study.

Patients with CoA more often have hypertension and hypercholesterolemia which predispose to coronary artery disease [6]. However, only 6 (former) smokers were included in our study and only one patient had an elevated cholesterol level. Therefore, no conclusions can be drawn regarding these risk factors and GLS.

### Relationships with conventional echocardiographic measurements

Although the anteroposterior LA diameter did not differ significantly from that of healthy controls, we observed that the LA diameter correlated with the GLS. This suggests LV–LA interaction which is in line with the arterial-LV–LA interaction observed in CoA patients [11], as well as in preclinical patients with cardiovascular risk factors [25]. An enlarged LA, even when measured only in anteroposterior direction, is associated with adverse cardiovascular outcomes [26]. In contrast, LA area did not correlate with GLS, possibly due to the poor image quality of the LA at the A4C. Furthermore, LV mass is an important risk factor for cardiovascular events [27]. In our study, a higher LV mass, but also thicker LV posterior wall and interventricular septum, both important parameters for LV mass, were associated with lower GLS. The patients of our study cohort were relatively young with a mean age of 33 years, and therefore surveillance of GLS might be regarded as an important treatment target in reducing the risk for events.

### Intra-observer and inter-observer agreement

The coefficients of variation for the intra-observer and inter-observer measurements were acceptable and comparable to other STE studies [28, 29].

### Limitations

A limitation is the relatively heterogeneous group of patients restricting the ability to perform some subgroup analyses. Significant differences we found between subgroups should be interpreted with caution.

## Conclusions

Despite a well-repaired CoA, the majority of adult patients have decreased LV GLS at late follow-up, while conventional 2D-echocardiography showed normal systolic LV function. Patients with higher blood pressure, associated congenital cardiac anomalies, higher LV mass or larger LA dimension have more decreased LV GLS. Whether decreased LV GLS will eventually lead to clinical heart failure and can identify patients in subclinical heart failure, and whether early detection can reduce morbidity, needs to be investigated in follow-up studies.

## Electronic supplementary material

Below is the link to the electronic supplementary material.

Supplementary material 1 (PDF 34 kb)
