# Supplementary material for: Quantitative assessment of systolic left ventricular function with speckle-tracking echocardiography in adult patients with repaired aortic coarctation
Source: Int J Cardiovasc Imaging. 2016 Jan 16;32:777–87. doi: 10.1007/s10554-016-0838-8 (PMC4853451; doi:10.1007/s10554-016-0838-8)
Supplement: Supplementary file 1 — Supplementary material 1 (PDF 34 kb) [file 10554_2016_838_MOESM1_ESM.pdf]

Online Resource 1. Clinical and echocardiographic characteristics stratified by global longitudinal strain values in tertiles

**Tertiles (GLS, %):**	**10.6-16.1%**	**16.2-17.9%**	**18.0-22.3%**	***P*-value**
	**n=23**	**n=22**	**n=23**	**Kruskal wallis**
Age (years)	37.1 [21.6-48.4]	27.8 [22.6-35.4]	30.2 [20.7-37.7]	0.394
Body mass index (kg/m^2)	26.3 [23.5-28.7]	24.4 [21.9-28.4]	22.8 [21.3-26.3]	0.097
Systolic blood pressure (mmHg)	131 [127-155]	131 [127-140]	135 [119-140]	0.583
Diastolic blood pressure (mmHg)	81 [75-90]	80 [74-85]	74 [67-79]	0.047
QRS duration (ms)	113 [103-138]	106 [97-118]	112 [104-119]	0.32
LA dimension (PLAX) (mm)	36 [32-41]	36 [30-42]	33 [30-35]	0.052
LV end-diastolic dimension (mm)	51 [46-55]	49 [47-53]	49 [45-53]	0.702
LV end-systolic dimension (mm)	31 [28-33]	31 [28-35]	31 [27-33]	0.812
Interventricular septum (mm)	10 [9-11]	9 [8-11]	9 [8-10]	0.018
LV posterior wall (mm)	10 [9-10]	9 [8-10]	8 [7-9]	0.023
LV mass calculated (g)	181.1 [146.9-214.2]	160.0 [138.3-210.7]	149.45 [120.2-175.0]	0.127
LV EF Simpson's (%)	54 [52-57]	56 [54-61]	61 [53-66]	0.032
LV deceleration time (ms)	185 [160-218]	191 [159-251]	201 [175-241]	0.784
LV E-top (m/sec)	0.87 [0.73-1.17]	1.00 [0.86-1.20]	1.10 [0.90-1.16]	0.092
LV E/A-ratio	1.33 [1.09-1.66]	1.49 [1.27-1.99]	1.58 [1.26-2.25]	0.161
LV E' (cm/sec)	8.11 [6.68-9.49]	9.80 [8.34-12.60]	8.89 [7.68-11.43]	0.035
NT-proBNP	9.4 [2.7-21.1]	7.2 [5.2-13.9]	5.5 [2.6-10.3]	0.475

Data are presented as median [1st quartile-3rd quartile].

E = peak mitral inflow velocity at early diastole; E' = early diastolic annular myocardial velocity; EF = ejection fraction; GLS = global longitudinal strain; LA = left atrium; LV = left ventricle; NT-proBNP = N-terminal pro-Brain Natriuretic Peptide; PLAX = parasternal long-axis view
